# Analysis of Food Production and Consumption Based on the Emergy Method in Kazakhstan

**DOI:** 10.3390/foods10071520

**Published:** 2021-07-01

**Authors:** Mengmeng Jia, Lin Zhen

**Affiliations:** 1 Institute of Geographic Sciences and Natural Resources Research, Chinese Academy of Sciences, 11A Datun Road, Chaoyang District, Beijing 100101, China; jiamm.19b@igsnrr.ac.cn; 2 College of Resources and Environment, University of Chinese Academy of Sciences, A19 Yuquan Road, Shijingshan District, Beijing 100049, China

**Keywords:** food production and consumption, emergy analysis, influencing factors, Kazakhstan

## Abstract

The imbalance in the supply and demand for resources is a thorny issue that many countries have to face. Food is a basic resource throughout the world. Understanding the exact situation of food production and consumption is an important foundation for sustainable development. This paper aims to explore the quantity and pattern changes in food production and consumption in Kazakhstan. This can reflect the level of residents’ standard of living and the situation of natural resource use. This paper focuses on the quantitative relationship between animal-based food and plant-based food and the tendency towards changes in urban and rural residents’ food production and consumption in Kazakhstan based on the emergy analysis method. The data of food production and consumption were mainly obtained from the official statistics of the Food and Agriculture Organization (FAO), the World Bank, Statistical Commission of the Ministry of National Economy of Kazakhstan, and literature. The research results showed that, over time, Kazakhstan residents’ food consumption patterns have become more varied, and the proportion of meat consumption to total food consumption has increased. Although the rural per capita food consumption is significantly different from that of urban residents, this gap decreased in 2019. In Kazakhstan, the consumption of several types of food still relies on imports. Food production and consumption are affected by economic, social, and ecological factors. The results of this research can provide scientific support for reasonable and sustainable production and consumption strategies in Kazakhstan.

## 1. Introduction

The sustainability of food production and consumption is the foundation of human survival and is vital for the development of specific countries and areas. The balance of food production and consumption plays a pivotal role in the development of a country and area, and it reflects not only the food production level but also the standard of local diets. However, as global challenges are becoming more serious, such as the rapidly expanding population, ecological destruction, and threats to food security, it is urgent to address these issues in order to achieve sustainable development [1,2,3,4].

The global population continues to increase dramatically, resulting in extreme pressure on land use and the environment. All of these issues have encouraged researchers to explore the soil’s capability and potential, and it has been found that different soils have different production levels [5,6]. In the following in-depth research, the main focus is on food production, specific animal food production, influencing factors [7], and food security [8]. The primary research on food consumption is mainly about family consumption [9]; influencing factors for food consumption [10]; food consumption patterns [11]; and consumption changes in different regions, such as central Asia [12], Latin-America [13], North-America [13,14], India [15], and China [16]. In the current research on food production and consumption, the methods used mainly contain mathematical statistics [17], the cereal-equivalent method [18], ecological footprint [19], life cycle assessment [20], and model methods such as principal component analysis [21]. With the development of the geographic information system (GIS), the mapping method is gradually being integrated into research methods [22,23]. In the current research, the study of food production and consumption still lacks a suitable and unique calculation standard with which to assess different kinds of food in order to obtain a clearer idea of the local situations of food production and consumption.

The 17 Sustainable Development Goals (SDGs) that were adopted by world leaders in 2015 offer a global agenda towards 2030; among these goals, SDG 2, “Zero Hunger”, needs sustainable agriculture in order to make it come true, especially sustainable production and consumption [24]. COVID-19 increased the challenges worldwide for poor people and their diets (FAO, 2020). In light of the pandemic’s effects on the food and agricultural sector, sustainable food production and consumption have become more important than before. Many researchers have recognized that controlling the principles of food production and consumption is necessary for a country, especially providing scientific references and guidelines for government managers when making suitable agriculture strategies and land use design.

Choosing the correct method to evaluate a food situation is an important foundation for policymakers. Current research is focused on either consumption or production and its influencing factors [11,16,25,26], and many researchers have studied some useful evaluation methods to calculate the quantity of food production or consumption in order to dig out the tendency or crucial change points; some typical research methods include ecological footprint [19,27,28,29], modeling [23,30,31], and household survey [26,32,33]. All of the methods above still have shortcomings that are difficult to overcome when applying them to different situations, such as ecology, economy, and society. Emergy analysis can solve the problem that different types of energy are difficult to compare and calculate, and can measure the true value of products and services of the natural ecosystem, social system, and economic system, and can analyze the results to realize the connection between theoretical research and decision-making applications.

Emergy is the available energy of one kind of (usually solar energy) required directly or indirectly to make a product or service [34,35], and emergy is measured in emjoules (sej). In general, emergy is formulated as solar emergy because the biosphere originates from solar emergy directly or indirectly, and emergy is measured in solar emergy (sej) [15]. The emergy evaluation distributes a value to products and services by turning them into equivalents of solar energy joules, which can be used as a common denominator [36]. Therefore, different forms of resources with different units can be measured and compared to each other in this way [37]. In a single product study, there were wheat [37], maize [38,39], and milk [40]; researchers used the emergy method to find the evaluation on sustainable production about wheat, maize, and milk. As to the agriculture system, the effect relationship between agriculture and other systems such as environment, land-use, and agriculture’s inner system [41,42,43,44].

Research on food production and consumption in Kazakhstan is extremely important in order to make use of the country’s natural resources and the distribution of food trade, improving the sustainability of food production and consumption, and assisting with scientific decision-making for agricultural development. However, research on Kazakhstan food mostly focuses on food consumption or improving the quantity of food [45,46,47,48]. The comprehensive study linking both production and consumption patterns and their changes, differences between urban and rural food consumption, and the driving factors, is rarely considered and launched. This research employed the emergy method to explore the changing trend of food production and consumption quantity in Kazakhstan, characters of per capita food consumption between urban and rural residents, and analyzed the social and economic influencing factors.

## 2. Materials and Methods

### 2.1. Study Area

Kazakhstan is located in Central Asia, and with an overall area of 2.72 × 10^6^ km^2^, covering latitudes from approximately 40° N to 55° N and longitudes from 50° E to 85° E it is the largest landlocked country in the world. The total population is 18.5 million (World Bank, NW Washington, DC 20433, USA, 2019), and the population density is 6.79 people/km^2^. Kazakhstan has faced some issues, such as high immigration at the start of its independence and economic retrogression, as well as a decrease in the total population from 1997 to 2002. Its population was 18.51 million in 2019, 3.66 million higher than that in 2002. The urban population was 18.51 million in 2019, and this was higher than the rural population of 10.65 million for the same year. The percentage of the urban population kept increasing, from 55.96% in 1997 to 57.54% in 2019, with an increase rate of 1.58%. Kazakhstan’s economy rapidly developed, as the GDP continuously grew from USD 6.04 billion in 1997 to USD 21.33 billion in 2019, and the GDP in 2019 was 2.53-fold higher than that for 1997; growth even reached 10.7% in 2006. Agriculture is the country’s primary industry and has made a great contribution to the primary industry GDP.

**Figure 1:**
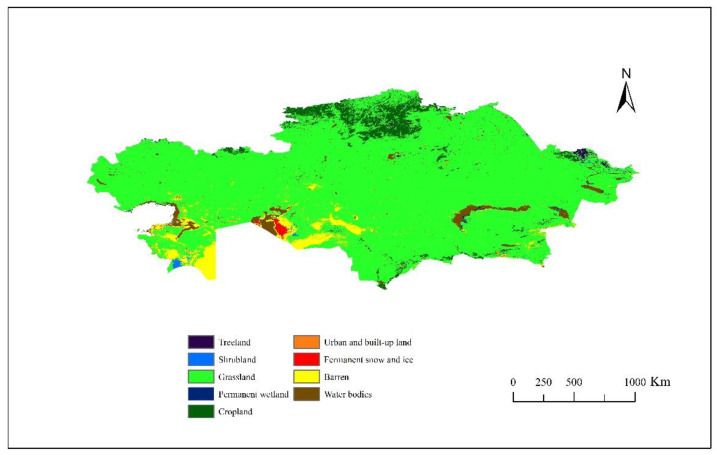
Land use in Kazakhstan in 2017.

The landform of the country is mainly plains and mountains. As it is located in the interior of the Eurasian landmass, it has a warm temperate continental climate and three climatic zones classified as arid, dry continental, and highland. The annual precipitation in the plains is about 100–300 mm, while in the southeastern mountains, it can rise to 800–1500 mm. As affected by the topography and precipitation, the main land-use types are farmland, grassland, and shrub from north to south [29]. Kazakhstan’s land use consists of cropland, grassland, forest, waterbody, barren, wetland, shrubland, urban and built-upland, and permanent snow and ice. The grassland area is 1.40 × 10^7^ km^2^, which accounts for 85.60% of the total land (Figure 1). Animal husbandry mainly consists of cattle, sheep, and poultry. Its primary animal products are beef, mutton, pork, chicken, milk, and eggs. The cropland area is smaller than the grassland, and it accounts for 5.40% of the total land. This kind of land mainly provides agricultural plants such as grains, beans, and potatoes; wheat accounts for half of the total agricultural land. In 2017, the proportion of agriculture in the total output value of the primary industry remained above 99%.

### 2.2. Data Collection

For this study, animal-based food includes beef, mutton, pork, chicken, fish, horsemeat, milk, and eggs; here, milk is from cows, and goat milk is excluded. Fish mainly includes freshwater and demersal fish. Plant-based food includes wheat, rice, potatoes, fruit, vegetables, and sugar. Fruit includes grapes, apples, and bananas, and vegetables include cabbage, carrots, cauliflower, broccoli, chilies, peppers, garlic, and eggplants. Food consumption data for Kazakhstan and its urban and rural residents per capita consumption from 1997 to 2019 were obtained from the official statistics of the Statistical Commission of the Ministry of National Economy of Kazakhstan (http://stat.gov.kz (accessed on 31 September 2020)). The food production data were derived from the Food and Agriculture Organization of the United Nations (FAO; http://www.fao.org/faostat/en/#data (accessed on 31 September 2020)). The size of the population, gross domestic production (GDP), trade, and other economic data were obtained from the World Bank (https://data.worldbank.org.cn (accessed on 31 September 2020)). Data on horsemeat consumption and the main factors that influence food production and consumption were obtained from social research of local residents in 2018. Land use data were extracted from the GADM database (www.gadm.org (accessed on 20 April 2018)), version 3.4, in April 2018.

### 2.3. Analysis Methodology

This work aimed to study Kazakhstan’s food production and consumption and their relationship from 1997 to 2019. According to the food source, the national foods were divided into plant-based and animal-based food. First of all, we needed to convert food production and consumption data into emergy as the specific food’s energy conversion rate and emergy transformity. The calculation of plant-based food production or consumption converted their production or consumption into emergy and summarized the food emergy; the specific calculation method is expressed in Equation (2). This showed plant-based food consumption as a trend in urban and rural areas. The calculation of animal-based food production or consumption is also expressed by Equation (2). This makes it possible to study the difference between animal-based food consumption for urban and rural residents. The food consumption of urban or rural residents is calculated using Equations (4) and (5). According to the studied urban and rural food consumption patterns, we tried to clearly find the change and its characteristics.

Emergy was used to calculate the total emergy. The equation expressing emergy comes from reference [49] (Table 1), and its detailed calculation is shown below.

(1) $$E_{i}=x_{i}\times k_{mi}\times k_{ni}$$

(2) $$E_{t}=E_{p}+E_{a}=\overset{h}{\underset{p=1}{\sum }}x_{p}\times k_{mp}\times k_{np}+\overset{h}{\underset{a=1}{\sum }}x_{a}\times k_{ma}\times k_{na}$$

(3) $$P_{t}=P_{u}+P_{r}$$

(4) $$E_{U}=P_{u}\times E_{t}$$

(5) $$E_{r}=P_{r}\times E_{t}$$

*i*: specific food, such as wheat, rice, potato, beef, mutton, pork, and chicken;

*a*: animal-based food;

*p*: plant-based food;

$x_{i}$: represents specific food quantity (kg);

$k_{mi}$: energy conversion rate (J/kg);

$k_{ni}$: emergy transformity (sej/J);

$E_{p}$: per capita annual plant-based food consumption (sej);

$E_{a}$: per capita annual animal-based food consumption (sej);

$E_{t}$: per capita annual food consumption (sej);

$P_{t}$: total population;

$P_{u}$: urban population;

$P_{r}$: rural population;

$E_{r}$: rural residents’ annual food consumption (sej);

$E_{U}$: urban residents’ annual food consumption (sej).

This paper focused on exploring the relationship between food production and consumption and both characteristics in Kazakhstan from 1997 to 2019 by applying the emergy analysis method. The specific food emergy transformity information is shown below, and the relative index is central Asia.

## 3. Results

### 3.1. Food Production and Consumption Quantity and Their Change Trend

With the continuous development of the economy and society, food production and consumption have undergone different changes in terms of quantity and patterns. According to the rising and falling trends of food consumption, food consumption in Kazakhstan can be divided into three stages: the subsistence consumption stage (1997–2000), the quality consumption stage (2001–2010), and the development consumption stage (2011–2019). At different consumption stages, the national, and urban and rural residents also show different characteristics in terms of food consumption quantity and patterns.

(1) Total food production and consumption increase and pattern change

**Figure 2:**
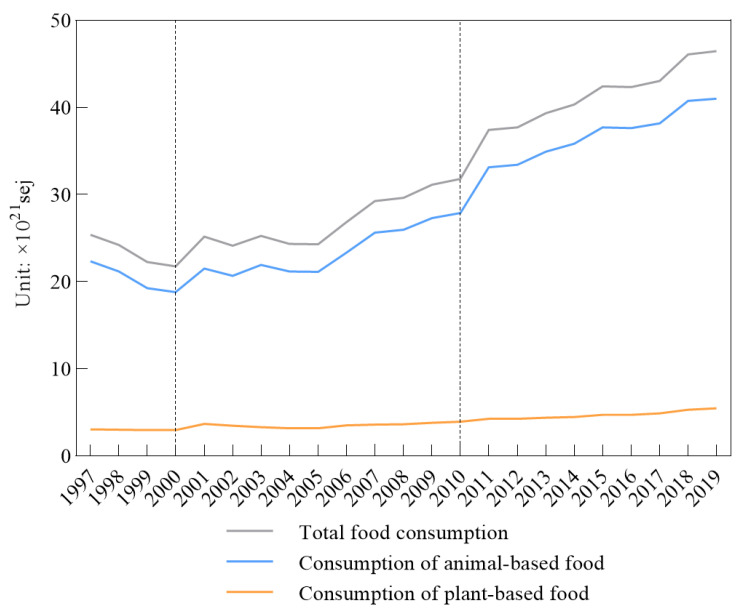
Annual food consumption in Kazakhstan.

Animal-based food consumption occupied the largest percentage and appeared to be growing (Figure 2); the average percentage of animal-based food consumption in the total food consumption was 87.64%. Although plant-based food consumption increased from 3.06 × 10^21^ sej in 1997 to 5.46 × 10^21^ sej in 2019, its growth rate was less than 3%; this kind of food consumption remained relatively stable.

**Figure 3:**
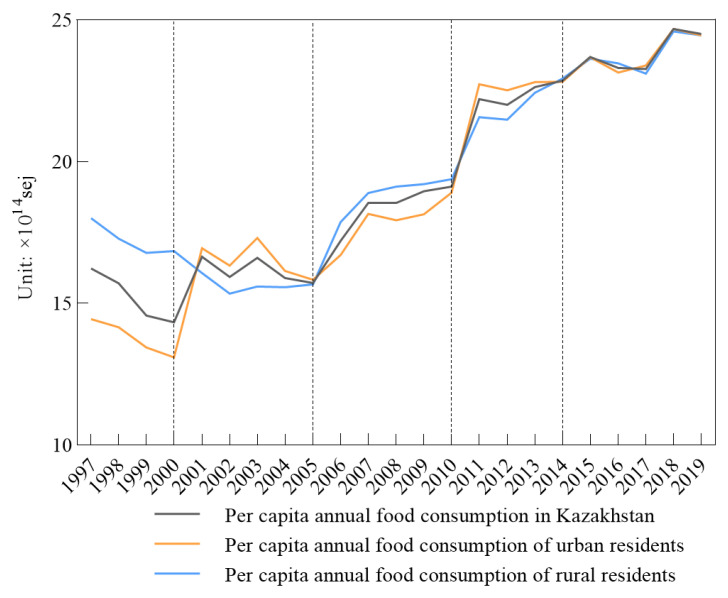
Per capita annual food consumption.

The per capita annual food consumption at the national, urban, and rural levels showed different results and kept fluctuating (Figure 3). Here, national food consumption was taken from both the urban and rural food consumption from 1997 to 2019. Urban food consumption was less than that for the rural residents from 1997 to 2000, and from 2006 to 2010, while the periods of 2001 to 2005 and 2011 to 2014 showed that urban food consumption was more than that for the rural residents. From 2015, the food consumption for national, urban, and rural residents gradually became more similar.

**Figure 4:**
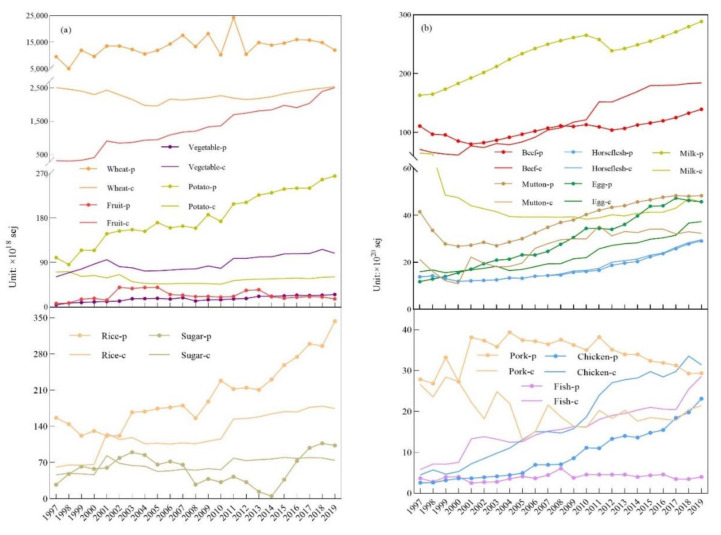
Annual food production and consumption in Kazakhstan. (**a**) Annual plant-based food production and consumption in Kazakhstan; (**b**) annual animal-based food production and consumption in Kazakhstan (-c represents consumption and -p represents production).

The production of plant-based food, namely, wheat, rice, and potatoes, exceeded consumption throughout our research years, and fruit and vegetable production was below consumption (Figure 4a). Compared with that for other plant-based food, the relationship between sugar production and consumption was diverse. The highest amount of sugar production and consumption was 71.71 × 10^18^ sej in 2014. Most of the animal-based food production was lower than the consumption, such as for chicken, fish, horsemeat, and beef. Egg production exceeded consumption since 2001. Milk, mutton, and pork production always exceeded the consumption for our research years (Figure 4b).

In Kazakhstan, the consumption of both plant-based and animal-based foods increased and decreased to different degrees. For plant-based food, fruit consumption had a significantly increased trend, and the same was seen for beef, but the increase in consumption for beef was smaller than that for fruit. From 1997 to 2019, wheat consumption fluctuated greatly; the highest wheat consumption was 2.54 × 10^21^ sej in 2019, and the lowest was 1.96 × 10^21^ sej in 2005; this means that wheat consumption achieved a 30% growth compared with 2005. In the subsistence consumption stage from 1997 to 2000, wheat consumption had a significant increase from 8.8 × 10^13^ sej in 1997 to 1.09 × 10^14^ sej in 2000, and the rate of wheat flour consumption decreased sharply from 6.34 × 10^13^ sej in 1997 to 2.96 × 10^13^ sej in 2000.

The consumption of other plant-based foods, such as rice, potatoes, vegetables, and sugar, remained at a relatively stable level without much change (Figure 4a). The annual output of plant food, especially wheat and other grains, fluctuated greatly, while the output of animal food showed an overall upward trend. Wheat production is greatly affected by local climate change, and the production fluctuated greatly; the lowest and highest wheat production was 5.07 × 10^21^ sej in 1998 and 2.43 × 10^22^ sej in 2011, respectively, and wheat production exhibited a 3.8-fold increase. The production of fruit and vegetables was low and stable; the production for both remained at an average of 2.32 × 10^19^ sej and 1.64 × 10^19^ sej from 1997 to 2019, respectively. The production of potatoes and rice showed a steady upward trend from 9.98 × 10^19^ sej and 1.56 × 10^20^ sej in 1997 to 2.65 × 10^20^ sej and 3.43 × 10^20^ sej in 2019, respectively. Potato production showed significant growth, with a 2.1-fold increase. In the same period (1997–2000), most animal-based foods, such as beef, mutton, and milk, were in decline, except pork. Fish and chicken remained constant. In the quality consumption stage, both plant-based food and animal-based food showed mostly stable increases. However, wheat, milk, and pork consumption showed a gradual decrease. In the development consumption stage from 2011 to 2019, fruit and beef maintained the same increase trend as for the quality consumption stage. Fruit consumption increased from 1.69 × 10^21^ sej in 2011 to 2.51 × 10^21^ sej in 2019, which was a 48% increase. Wheat and milk maintained a decreasing trend, and other foods, such as rice, sugar, vegetables, and potatoes, maintained a low and stable consumption. Chicken, egg, and fish showed a small margin of increase. All animal-based food production showed different increases from 1997 to 2019. Pork and fish production showed a slight upward trend and fluctuation that increased from 2.79 × 10^21^ sej and 3.67 × 10^20^ sej in 1997 to 2.94 × 10^21^ sej and 3.98 × 10^20^ sej in 2019, respectively. Beef and milk production had a significant increase from 1.12 × 10^22^ sej and 1.63 × 10^22^ sej in 1997 to 1.39 × 10^22^ sej and 2.89 × 10^22^ sej in 2019, respectively, but there was little fluctuation for beef and milk production, while other items showed an upward trend to different degrees. Chicken and egg production also showed a small upward trend, although the growth margin was not as large as for beef and milk. Chicken and egg production was mainly slow and steady, from 2.58 × 10^20^ sej and 1.18 × 10^21^ sej in 1997 to 2.31 × 10^21^ sej and 4.58 × 10^21^ sej in 2019, respectively.

**Figure 5:**
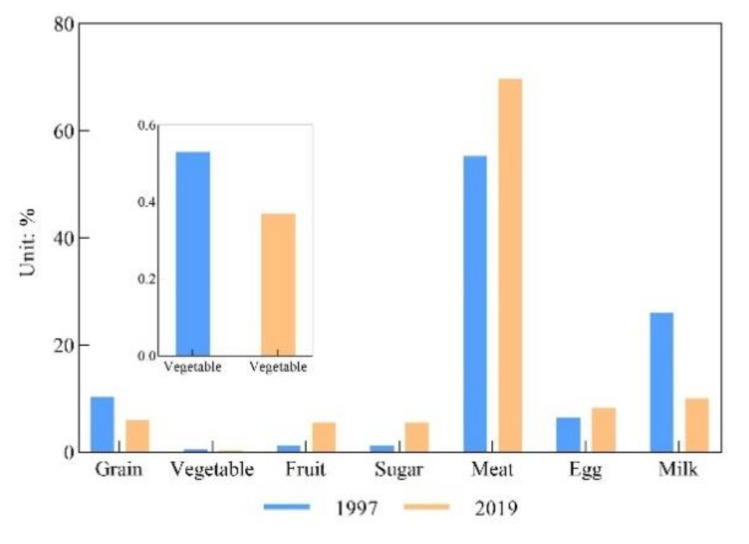
Food consumption patterns in Kazakhstan in 1997 and 2019.

For the food consumption pattern, food consumption items remained stable and did not change much; the significant change was in the meat percentage of food consumption over the 23 years (Figure 5). Comparing the food consumption percentage from 1997 with 2019, the results showed that meat consumption accounted for 69.68% of the total food consumption and had a 15.18% increase over 23 years. The fruit consumption percentage also had a great increase of 4.27% from 1997 to 2019. At the same time, the egg consumption percentage had a relatively low increase of 1.69%. In contrast to meat and fruit, grains and milk decreased by 4.55% and 16.56%, respectively, from 1997 to 2019. Sugar and vegetable consumption remained stable, as they showed slight decreases of 0.02% and 0.01%, respectively.

### 3.2. Changing Relationship between Food Production and Consumption

The total food production met the domestic food consumption, but the supply of some food was insufficient, especially for plant-based food, mainly fruit and vegetables, and animal-based food, mainly chicken and fish, which constantly showed that consumption exceeded production, and the gap between consumption and production for these four kinds of food was constantly increasing.

**Figure 6:**
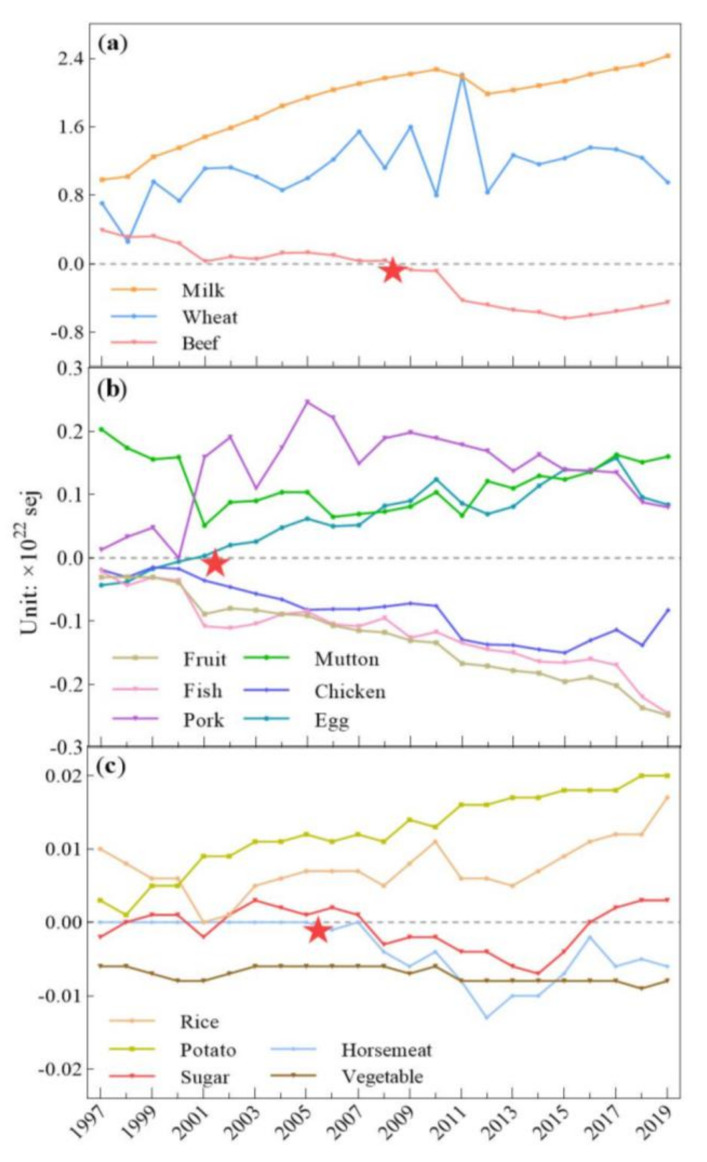
Annual food gap between local production and consumption in Kazakhstan. (★ represents the turning point). (**a**) annual local production and consumption gap of milk, beef and wheat; (**b**) annual local production and consumption gap of fruit, fish, pork, mutton, chicken and egg; (**c**) annual local production and consumption gap of rice, potato, sugar, horsemeat and vegetable.

Production exceeding consumption means that the domestic food supply can satisfy domestic consumption. The kinds of food that have always been in oversupply are mainly wheat, rice, potatoes, milk, mutton, and pork (Figure 6a–c). Wheat production always exceeded consumption, and the biggest gap for this reached 2.21 × 10^22^ sej, in 2011, while it was the lowest, at 2.61 × 10^21^ sej, in 1998 (Figure 6). In 2011, this quantity was 7.47 times more than that in 1998, meaning that the growth in wheat production greatly exceeded that of its consumption (Figure 6a). The biggest gap between the production and consumption of potatoes was 2.04 × 10^20^ sej in 2019, and the smallest was 1.42 × 10^19^ sej in 1998. The difference between these two gaps was 13.4 times. Milk production always exceeded its domestic consumption, with the biggest gap being 2.43 × 10^22^ sej in 2019 and the smallest one being 9.83 × 10^21^ sej in 1997. The production and consumption gap of milk in 2019 was 1.47 times more than that in 1997. The biggest and smallest gap between the production and consumption of mutton was 2.03 × 10^21^ sej in 1997 and 6.67 × 10^20^ sej in 2011; the gap in 2011 was 2.05 times less than that in 1997. Pork consumption in Kazakhstan was relatively less than that for countries such as China. The gap where pork production could not meet its consumption only occurred in 2000, with a gap of 4.95 × 10^18^ sej. The biggest gap for pork was 2.46 × 10^21^ sej in 2005. The smallest and biggest gap between the production and consumption of rice was 7.27 × 10^18^ sej in 2001 and 1.69 × 10^20^ sej in 2019, respectively. The gap in 2019 was 22.2 times more than that in 2001.

There are still several kinds of food where production falls short of consumption, such as plant-based food, namely, fruit and vegetables, and animal-based food, namely chicken and fish (Figure 6b,c). Fruit and vegetable production did not meet domestic consumption, and still, 1.28 × 10^21^ sej and 7.20 × 10^19^ sej imports were demanded, respectively, from 1997 to 2019. Fruit and vegetables’ biggest gaps between production and consumption were 2.49 × 10^21^sej in 2019 and 9.27 × 10^19^ sej in 2018, respectively. For plant-based food, the production of domestic fruit and vegetables did not satisfy local residents’ consumption, which is becoming a serious issue—the production/consumption of fruit expanded from 5% in 1999 to 1% in 2019, and the fruit gap between local production and domestic consumption changed from 3.09 × 10^20^ sej in 1997 to 2.49 × 10^21^ sej in 2019. The gap between vegetable production and consumption also increased from 5.65 × 10^19^ sej in 1997 to 8.34 × 10^19^ sej in 2019. Fish production was in deficiency, and the average gap remained at a low level of 3.28 × 10^20^ sej from 1997 to 2000. From 2001, the fish production deficiency increased, and the biggest gap was 2.46 × 10^21^ sej in 2019. The redundancy of rice and potatoes was 1.25 × 10^20^ sej and 5.61 × 10^19^ sej on average. The chicken production and consumption gap presented continuous growth. The smallest and biggest gaps between chicken production and consumption were 1.71 × 10^20^ sej in 2000 and 1.50 × 10^21^ sej in 2015. The difference between the gap in 2015 and in 2000 was 7.76-fold.

**Figure A1:**
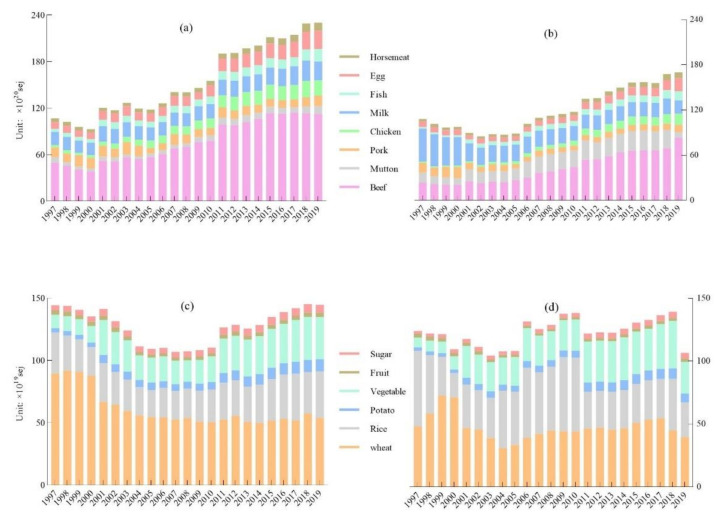
Annual animal-based and plant-based food consumption of urban and rural residents. (**a**) Annual animal-based food consumption of urban residents; (**b**) annual animal-based consumption of rural residents; (**c**) annual plant-based food consumption of urban residents; (**d**) annual plant-based food consumption of rural residents.

During the study period, several kinds of food production and consumption gaps showed turning points. This category of food contained horsemeat, eggs, and beef. Horsemeat consumption is a famous local food in Kazakhstan. Its production satisfied its domestic consumption during the first three years from 1997 to 2005. However, from 2006, the turning point came when the horsemeat supply could not meet the domestic demand. In 2006, urban and rural horsemeat consumption showed increases of 7.18% and 6.86%, respectively, compared with that of the previous year, and this consumption growth continued to increase (Appendix A Figure A1a,b), with the gap gradually getting larger until its peak of 1.05 × 10^20^ sej in 2013.

Both beef and egg production and consumption had turning points, which means that the gap direction between production and consumption changed. In 2009, the gap of beef production and consumption reached a turning point; the gap turned from positive to negative, which means that beef production could not remain self-sufficient, and imports from other countries were needed to satisfy the domestic beef demand. Both urban and rural beef consumption grew at high speed, especially in 2011, where urban and rural beef consumption reached 9.89 × 10^21^ sej and 5.42 × 10^21^ sej, which was a 27.31% and 22.45% increase compared with that of 2010 (Figure A1a,b). This contributed to the beef gap becoming larger. Beef’s biggest gap was 6.38 × 10^21^ sej in 2015. Compared with those for beef and horsemeat, the trends of change in egg production and consumption were different. Egg production could not satisfy domestic consumption during the period of 1997 to 2000. From 2001, the egg production and consumption gap turned from negative to positive and kept growing until it reached its peak of 1.58 × 10^21^ sej in 2017, which means that egg production was self-sufficient and met domestic consumption.

## 4. Discussion

During the study period, different food gaps between production and consumption presented various trends. Food production and consumption quantity and pattern evolution are affected by many different factors, such as economic, social, and ecological factors. When searching for the causes of the trends, we found that one of the most popular reasons was population change, which has been previously mentioned [50]. Since 2000, the total food consumption increased to 46.49 × 10^21^ sej, which is 2-fold more than that in 2000 (Figure 2). The population change trend was similar to the total food consumption and had continuing growth from 14.86 million persons in 2001 to 18.04 million persons; although the population explained the food consumption change independently, the total food consumption volume relied on the population quantity.

GDP represents one country or area’s comprehensive economic ability. This factor has a significant role in improving residents’ food consumption quantity and quality, especially in rural areas [51,52]. The GDP in Kazakhstan showed high growth from 1997 to 2019. GDP growth provides a strong foundation for people to purchase more and more varied foods. The final consumption expenses of a household’s disposable income also prove this point. The factor that plays a big role in food consumption quality is per capita final consumption expenses of the household disposable income, and related research has certificated this result [53]. In Kazakhstan, urban and rural meat consumption as a proportion of the total food consumption increased greatly from 64.06% and 45.46% in 1997 to both being 68.91% in 2019.

When the total population increases, urbanization in Kazakhstan also grows, and it grew from 55.96% in 1997 to 57.54% in 2019, which required more food types and put more pressure on land use [54]. From 1997 to 2019, Kazakhstan’s agricultural land area experienced decreasing firstly and then increasing, comparing with the least agricultural land area of 2.11 × 10^8^ ha, it grew by 3.03% in 2010, and since then it was kept at 2.16 × 10^8^ hm^2^ on average. In the meantime, cropland area also increased to 2.99 × 10^7^ hm^2^ in 2018, which was 4.97% more than that in 2003. More food consumption stimulated more land use for production [55,56]. Consumption structure change took adjustment of land use types [57]. With the improvement in the living standard over the past years, the consumption of non-staple foods such as vegetables and oilseed increased, leading to changes in land use structure. Oilseed plants and vegetable acreage increased to 2.86 × 10^6^ hm^2^ and 1.59 × 10^5^ hm^2^ in 2019, which were 7.58 folds and 82.66% more than that in 1997, respectively. Kazakhstan’s land is not fertile, and its ecological environment is relatively fragile [58,59]. Crop production and yield need to grow faster than the population and urbanization, which requires a sustainable approach. An increased urbanization rate means that the urban population is increasing, and more high-quality food is needed, such as meat. Meat consumption for the urban area went from 8.90 × 10^14^ sej in 1997 to 1.62 × 10^15^ sej in 2019, with a growth rate of 81.60%. The meat consumption increase meant more feed crop production was required, and this caused a great change in land use [60]. At the same time, more greenhouse emissions arose from agricultural production, and this also has an impact on the natural environment [61].

**Figure A2:**
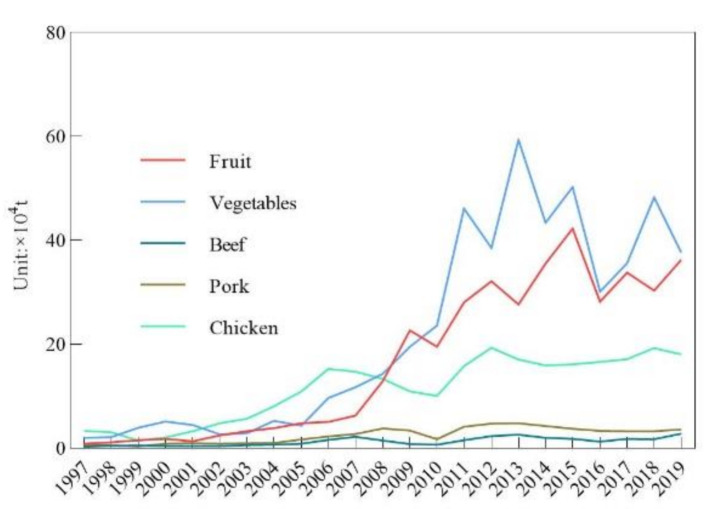
Food import quantity.

In order to meet the domestic food demand and ensure a decrease in cost, many countries select food import as a suitable way to transfer the production pressure to other countries [62]. In Kazakhstan, fruit and chicken imports improved greatly from 5.15t × 10^3^t and 3.31t × 10^4^t in 1997 to 2.83 × 10^5^t and 1.71 × 10^5^t in 2019, both of which had large growth, which showed that the dependence on fruit and chicken imports increased. Vegetable imports also presented a great increase to 3.77 × 10^5^t in 2019, which was 18.21 folds that in 1997. Although domestic beef and pork production both improved, these two kinds of food still needed some import of 1.23 × 10^3^t and 2.24 × 10^3^t on average from 1997 to 2019 (Figure A2). However, this is also a food security risk, especially during the anti-globalization and worldwide pandemic era, which prohibited world trade connection and produced more isolation [62,63].

The ecological environment is the basic foundation of human life and provides many various foods [50,64]. Different food yields and livestock quantities are guaranteed in the usual food supply. Kazakhstan’s plant-based food focuses on staple foods such as wheat and rice; it is especially famous for its wheat production and export in the world. Agriculture is the basic industry in Kazakhstan, and the government focuses greatly on this. The yields of several foods have shown increases to different degrees. The potato yield rapidly increased from 8.41 × 10^4^ kg/hm^2^ in 1997 to 20.34 × 10^4^ kg/hm^2^ in 2019. This was the main reason Kazakhstan’s local potato could meet the domestic consumption of potatoes, and both the urban and rural potato consumption increased from 3.31 × 10^19^ sej and 2.73 × 10^19^ sej in 1997 to 9.65 × 10^19^ sej and 7.12 × 10^19^ sej in 2019, respectively. Animal-based food consumption always makes up a large portion of the usual food. According to the data of the food consumption patterns of Kazakhstan residents, the data shows that animal-based food per capita consumption accounted for 69.49% in 2019, increasing at a rate of 15.18% compared with that of 1997. Meat consumption was a large portion of the total food consumption, partly because the livestock quantity increased, especially chicken, sheep, and cattle stock, from 15.30 × 10^6^ head, 12.87 × 10^6^ head and 5.42 × 10^6^ head in 1997 to 43.42 × 10^6^ head, 16.91 × 10^6^ head, and 7.44 × 10^6^ head in 2019, respectively. Pig stock quantity showed a slight increase, and it also met Kazakhstan’s domestic pork consumption of 2.03 × 10^21^ sej on average.

## 5. Conclusions

According to the results above, Kazakhstan’s food production and consumption significantly increased in both quality and quantity. Animal-based food consumption showed a continued increase, especially for beef and horsemeat, and both of their production and consumption gaps turned from positive to negative. The consumption of plant-based food, such as wheat and rice, presented a decrease. Fruit and vegetable consumption continued to exceed production, and the gap continued to grow. In order to address these food insufficiencies, Kazakhstan has to depend on imports from other countries.

Kazakhstan’s residents’ food consumption had a significant trend showing that animal-based food consumption consisted of the highest total per capita food consumption (Figure 4). The Kazakhstan food consumption pattern changed with social and economic development. The significant change was that grain consumption as a proportion of the total food consumption was decreasing. The annual grain consumption in the urban and rural areas decreased from 11.46% and 10.07% in 1997 to 5.39% in 2019. Urban grain consumption decreased more than for rural residents (Figure A1c,d). Meat annual consumption as a proportion of the total food consumption increased from 64.06% in 1997 to 68.91% in 2019, but that of rural residents increased from 45.46% in 1997 to 68.91% in 2019. This shows that rural meat consumption is more vulnerable to social and economic influence than urban meat consumption, to some extent. Moreover, Kazakhstan has a large area of cropland and grassland, but it is easy to understand that its yield does not show a high efficiency for cropland utilization because its famous crop, wheat, has a yield lower than that in Russia [65]. Compared with the international wheat yield, Kazakhstan’s wheat yield still has much room and potential for improvement. The yield level of crops in Kazakhstan had a long-term unstable situation, especially wheat and other cereals. Thus, it is important to implement advanced plant technology and prepare for a decrease in yield brought about by climate change.

The per capita food consumption gap between rural and urban residents is getting smaller; this means that food consumption in Kazakhstan is converging. In 2019, the per capita annual food consumption of urban and rural residents was inclined to be equal. Kazakhstan’s local food production cannot satisfy all of the food consumption. Fruit, vegetables, beef, chicken, and fish consumption need to depend on imports from other countries, and the import quantity is increasing. Kazakhstan has to improve these kinds of local food productions in order to reduce the dependence on food imports. The COVID-19 pandemic situation is still serious and has seriously affected the worldwide trade, including food imports [66,67,68]. More attention needs to be given to the improvement of agriculture technology and management efficiency. This will provide great help to Kazakhstan by implementing various measures to achieve more domestic food production.

As the population increases and economic development stimulates more food consumption and various types of food production in Kazakhstan, the pressure on land use and the environment is increasing. Therefore, the establishment of sustainable food production and consumption will contribute to ensuring harmony between human beings and nature.

This research revealed the food production and consumption patterns in Kazakhstan; it still has some limitations: first, lack of sufficient data on the state-wide production and consumption that can be used to investigate the spatial variations; meanwhile, food waste data is not available and thus limited us from understanding the gaps between production and consumption; second, the influence of residents’ education and culture on their consumption needs to be explored through interviews or literature reviews. Third, we used average conversion factors in central Asian countries to convert food production and consumption data into emergy for Kazakhstan; this may have affected, to some extent, the accuracy of the results. In this regard, the research team will employ online or face-to-face interviews and other useful methods to gather necessary data and information to support further studies.

## Figures and Tables

**Table 1:** Energy conversion rate and emergy transformity for each food item [49].

Item	Energy Conversion Rate (J/kg)	Emergy Transformity (sej/J)
Wheat	1.57 × 10^10^	6.80 × 10^4^
Rice	1.55 × 10^10^	3.95 × 10^4^
Potato	2.51 × 10^9^	2.70 × 10^4^
Vegetable	2.51 × 10^9^	2.70 × 10^4^
Fruit	3.30 × 10^9^	5.30 × 10^5^
Sugar	2.50 × 10^9^	8.50 × 10^4^
Beef	8.76 × 10^9^	3.17 × 10^6^
Mutton	1.41 × 10^10^	2.00 × 10^6^
Pork	2.00 × 10^10^	1.70 × 10^6^
Chicken	5.40 × 10^9^	2.00 × 10^6^
Milk	2.90 × 10^9^	1.71 × 10^6^
Horsemeat	1.10 × 10^7^	2.00 × 10^6^
Fish	5.40 × 10^9^	2.00 × 10^6^
Egg	8.30 × 10^9^	2.00 × 10^6^

## Data Availability

Not applicable.
